# Pulmonary sclerosing hemangioma in a 21-year-old male with metastatic hereditary non-polyposis colorectal cancer: Report of a case

**DOI:** 10.1186/1477-7819-9-62

**Published:** 2011-06-06

**Authors:** Tobias S Schiergens, Philipe N Khalil, Doris Mayr, Wolfgang E Thasler, Martin K Angele, Rudolf A Hatz, Karl-Walter Jauch, Axel Kleespies

**Affiliations:** 1 Department of Surgery, University of Munich, Campus Grosshadern, Germany; 2 Department of Surgery, University of Munich, Campus Innenstadt, Germany; 3 Department of Pathology, University of Munich, Munich, Germany

**Keywords:** Sclerosing hemangioma, Pneumocytoma, Colorectal cancer (CRC), Hereditary non-polyposis colorectal cancer (HNPCC), Lynch syndrome, Familial adenomatous polyposis (FAP)

## Abstract

**Background:**

Pulmonary sclerosing hemangioma (SH) is a rare tumor of the lung predominantly affecting Asian women in their fifth decade of life. SH is thought to evolve from primitive respiratory epithelium and mostly shows benign biological behavior; however, cases of lymph node metastases, local recurrence and multiple lesions have been described.

**Case Presentation:**

We report the case of a 21-year-old Caucasian male with a history of locally advanced and metastatic rectal carcinoma (UICC IV; pT4, pN1, M1(hep)) that was eventually identified as having hereditary non-polyposis colorectal cancer (HNPCC, Lynch syndrome). After neoadjuvant chemotherapy followed by low anterior resection, adjuvant chemotherapy and metachronous partial hepatectomy, he was admitted for treatment of newly diagnosed bilateral pulmonary metastases. Thoracic computed tomography showed a homogenous, sharply marked nodule in the left lower lobe. We decided in favor of atypical resection followed by systematic lymphadenectomy. Histopathological analysis revealed the diagnosis of SH.

**Conclusions:**

Cases have been published with familial adenomatous polyposis (FAP) and simultaneous SH. FAP, Gardner syndrome and Li-Fraumeni syndrome, however, had been ruled out in the present case. To the best of our knowledge, this is the first report describing SH associated with Lynch syndrome.

## Background

Sclerosing hemangioma of the lung (SH), alternatively characterized as alveolar pneumocytoma, was first described by Liebow and Hubbel in 1956 [1] and represents a rare and, in the majority of cases, benign neoplasm of the lung. It predominantly affects females in their fifth decade of life [2,3] and is more common in Asian women. Although several theories have been proposed for its histogenesis and the term implies an endothelial derivation, an origin from immature respiratory epithelium is currently accepted [3-7]. Symptoms such as atypical thoracic pain, cough, hemoptysis and dyspnea might occur due to tumor enlargement and compromising of surrounding tissue [3]. However, in most patients, SH is detected incidentally during routine chest radiographic examination because it is generally asymptomatic [2,8]. Although SH is thought to be benign, cases of lymph node metastases, local recurrence and multiple lesions have been reported [2,9-11] suggesting that the progression to an overtly malignant phenotype might be possible. Lymph node metastases, however, do not seem to have an impact on long-term survival [12]. Altogether, little is known about the associated risk factors, prognosis and natural course of SH, and little clinical data exists from western countries.

Only a few cases have been reported affecting young patients. There are two recent reports describing middle-aged female patients suffering from familial adenomatous polyposis (FAP) and simultaneous SH that suggest a common tumorigenesis and report SH as a part of the clinical phenotype of FAP [13,14]. Many hereditary syndromes associated with colorectal cancer (CRC) can have extracolonic manifestations. However, to the best of our knowledge, we present the first case of a patient with the diagnosis of SH and a history of Lynch syndrome.

## Case Presentation

We first diagnosed a 21-year-old Caucasian male suffering from CRC in January of 2009. The patient complained of having recurrent rectal bleeding for three months. He was otherwise a healthy non-smoker and in good condition appropriate for his age. His medical history was uneventful. Evaluation of family history revealed five relatives afflicted with malignant tumors at a young age. Among them were his mother, who died at the age of thirty-five from endometrial cancer, and the mother's brother, who passed away at the age of forty from CRC. The patient did not report significant weight loss, fever or night sweats. Physical examination was unremarkable. Carcinoembryonic antigen (CEA) and carbohydrate antigen 19-9 (CA 19-9) were within normal range. Clinical staging diagnostics revealed a partially stenosing rectal adenocarcinoma (uT4, uN+) but no potentially metastatic lesions in the liver or lung at that time. There was no clinical evidence of FAP or Gardner syndrome. Li-Fraumeni syndrome was subsequently ruled out by sequencing of multiple TP53-exons (3-9) after PCR amplification of genomic DNA.

With respect to locally advanced tumor growth, the patient underwent neoadjuvant 5-fluorouracil-based chemoradiotherapy (5-fluorouracil/folinic-acid, 50.4 Gy) followed by low anterior resection including total mesorectal excision in the spring of 2009. Intraoperative sonography of the liver showed a small lesion in segment VII, but, due to the locally advanced tumor stage (pT4, pN2 (6/9), uM1 (hep), V1, L1, G2, R0), we decided in favor of non-simultaneous resection of the hepatic lesion [15]. According to revised Bethesda guidelines [16], microsatellite instability (MSI) testing was performed by DNA isolation and subsequent PCR amplification from tissue of the primary rectal carcinoma resulting in detection of significant instability in microsatellites BAT25, BAT26, D17S250 and D2S123. This finding shaped up as high level of MSI (MSI-H). Moreover, sequencing of the protooncogenes *KRAS *and *BRAF *showed no mutation (wildtype). This raised the strong suspicion of a Lynch syndrome particularly with regard to the patient's family history, his age and the fulfillment of the Amsterdam criteria [17,18]. MSI in CRC of patients under the age of forty are estimated to be due to an underlying germline mutation in 85.7% of the cases, a probability, which is elevated by the presence of a *BRAF*-wildtype. The latter can be used to distinguish sporadic MSI CRC from MSI tumors that arise in the setting of Lynch syndrome [19]. Consecutively, the patient underwent human genetic counseling followed by testing for germline mutations in mismatch repair (MMR) genes by sequencing of their cDNA emanating from PAX-RNA and total RNA isolated from short-term lymphocyte culture. Thereby a mutation was detected in MMR-gene PMS2 (exon 11). Altogether, the diagnosis of Lynch syndrome was made.

**Figure 1:**
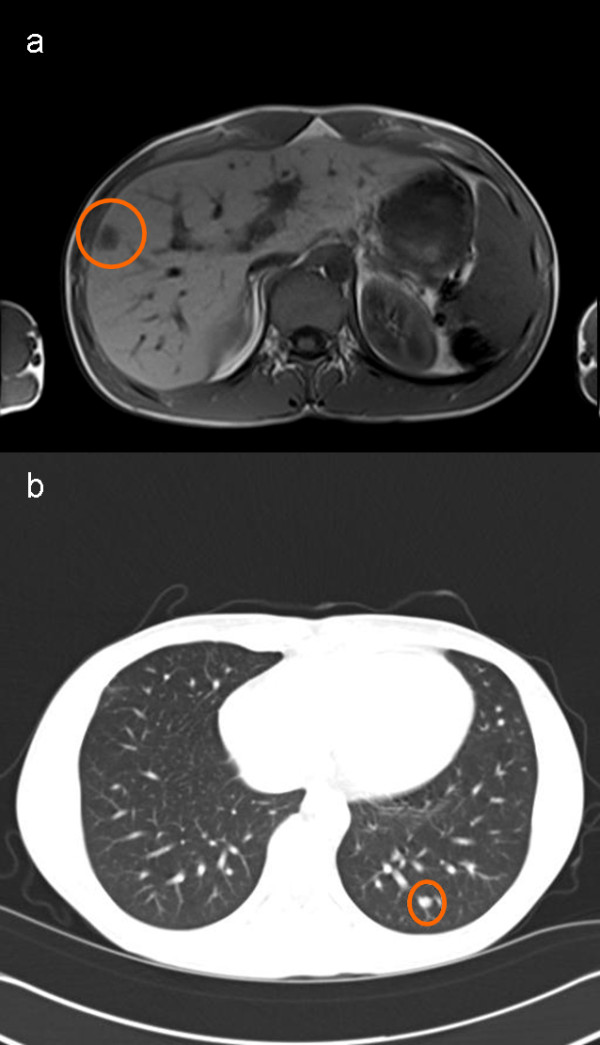
**Diagnostic imaging**: a) Magnetic resonance imaging (MRI) of the patient showing a colorectal liver metastasis in segment VII of the liver (*circle*) prior to its resection. b) Thoracic computed tomography exhibits a potentially metastatic, well-circumscribed lesion of 6 mm in the left lower lobe (*circle*) with homogenous contrast media enhancement. Pathological evaluation revealed a sclerosing hemangioma of the lung.

**Figure 2:**
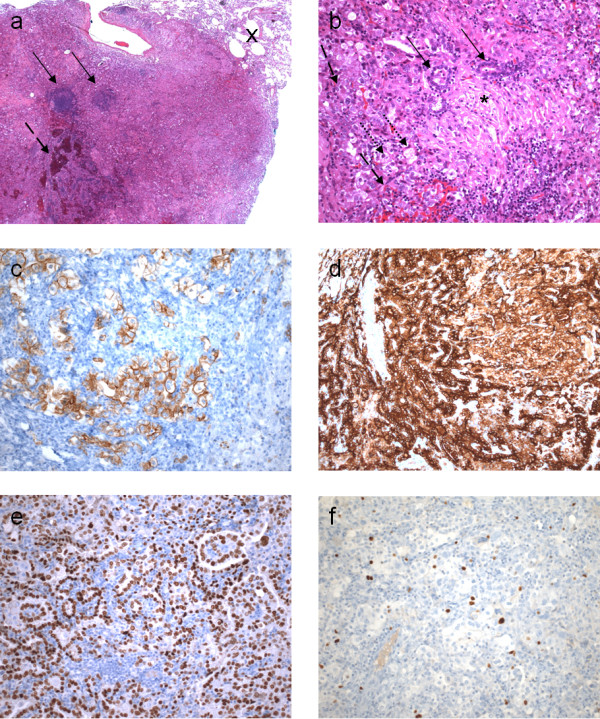
**Histology (a, b) and immunohistochemistry (c-f) of sclerosing hemangioma of the lung**: a) Well-circumscribed lesion with normal lung tissue in the right upper corner (X), lymphoid cell infiltration (arrows) and hemorrhages (dashed arrow); hematoxylin and eosin stain (25x) b) Mixed growth pattern of the lesion, papillary (arrows), solid (dashed arrows) and sclerotic (*); foam cells (dotted arrows); hematoxylin and eosin stain (200x) c) Cuboidal surface cells positive for staining with pan-cytokeratin antibody (200x) d) Epithelial membrane antigen (EMA) and e) thyroid transcription factor 1 (TTF-1) are positive in both cuboidal surface cells as well as stromal round cells (200x) f) Positive nuclear staining for Ki67 in only few cells (Ki-67-Index < 5%) (200x).

Early restaging was performed during intermittent FOLFOX chemotherapy and the patient was found to have hepatic (Figure 1a) and pulmonary lesions suspicious for metastases. Thoracic computed tomography showed a well-circumscribed 6 mm lesion in the left lower lobe of the lung (Figure 1b) with homogenous contrast media enhancement as well as two smaller lesions in the right upper lobe. There were neither signs of infiltration of the adjacent tissue nor signs of pathologically enlarged lymph nodes. We decided to first perform a partial hepatectomy (segment VII), which confirmed hepatic spread of the tumor. In the light of the patient's young age, his early recovery and his good general state of health, we proceeded to remove the left-sided pulmonary lesion four weeks later. Therefore, he underwent atypical resection of the left lower lobe through a left anterolateral thoracotomy followed by a systematic mediastinal and hilar lymphadenectomy [20]. The patient's postoperative course remained uncomplicated and he again recovered well. Gross examination of the specimen, however, showed a well-circumscribed solid pulmonary tumor, 7 mm in diameter. Histological evaluation revealed a mixed papillary, hemorrhagic and sclerotic growth pattern of cuboidal surface cells and polygonal stromal cells. Cuboidal surface cells were immunopositive for thyroid transcription factor-1 (TTF-1), epithelial membrane antigen (EMA) and pan-cytokeratin, whereas polygonal stromal cells were immunopositive for neuron-specific enolase (NSE) and S-100 protein as well as EMA. These findings are consistent with a sclerosing hemangioma of the lung (Figure 2). *Ki*-67 index was less than 5%. Both significant MSI evaluated by PCR amplification and loss of expression of MMR-proteins MLH1, MSH2, MSH6 and PMS2 determined by immunohistochemistry could not be detected in the pulmonary SH. Moreover, all lymph nodes sampled were free of metastases.

By thoracic computed tomography, the pulmonary lesions in the right upper lobe remained unchanged after 3 months. According to interdisciplinary tumor board recommendations and oncological guidelines [21-23] we decided not to suggest further chemotherapy or restorative proctocolectomy but to perform careful aftercare with monitoring of the pulmonary lesions at close intervals as well as attentive follow-up via abdominal ultrasound and colonoscopy.

Furthermore, the patient's family members were referred to cancer genetics specialists for counseling interviews and recommended germline mutation analysis. During regular follow up visits CEA and CA 19-9 were within normal range. Accurate colonoscopy and diagnostic imaging of liver and lungs were unremarkable, in particular pulmonary lesions of the right upper lobe both were not identifiable any more.

## Discussion

Pulmonary SH is a rare and mostly benign neoplasm of the lung. Histologically, SH is essentially characterized by two epithelial cell types: cuboidal surface cells, which resemble type II pneumocytes, and polygonal stromal cells (round cells) with bland nuclei and pale cytoplasm, which are thought to stem from primitive respiratory epithelium [4,5]. These two cell types form four histological patterns; papillary, which often appears to be the predominant type, but epitheloid, sclerotic and hemorrhagic configurations are also found in some cases as in the present one (Figure 2, [24]). Predominant papillary growth patterns might make it complicated to differentiate SH from a carcinoma that also exhibits a papillary pattern. Metastatic papillary thyroid carcinoma, mesothelioma and bronchioloalveolar carcinoma have to be considered accurately [11]. In this respect, however, decreased *Ki*-67 labeling and low p53 expression could help to differentiate SH from papillary thyroid carcinoma [2]. The cuboidal surface cells of SH are typically immunopositive for thyroid transcription factor-1 (TTF-1), epithelial membrane antigen (EMA), surfactant protein B (SP-B), low molecular weight cytokeratin (CK-L) as well as carcinoembryonic antigen (CEA) and negative for neuroendocrine markers, whereas polygonal stromal cells (round cells) are positive for vimentin and TTF-1 and weakly positive for several neuroendocrine markers [4,7,25]. Mitotic figures are rarely identified [2]. In the present case, the patient's lesion comprised mixed papillary growth patterns consisting of superficial layers of cuboidal cells that were immunopositive for TTF-1 and EMA, as well as stromal cells positive for TTF-1 expression, and some also for neuroendocrine markers such as neuron-specific enolase (NSE) and S-100 protein. Thus, histological and immunohistochemical diagnosis of SH was made, and a very low *Ki*-67 index of less than 5% indicated a biologically non-active tumor [26].

In most patients, SH is detected during routine chest radiographic examination [2,8]. Therefore, the actual prevalence of SH is not known due to the relatively asymptomatic nature of the disease. SH is usually diagnosed as a single asymptomatic nodule in the periphery of the lung [2,8], often affecting the lower lobe [27,28]. Radiologically, it mostly presents as a well-circumscribed lesion with marked contrast media enhancement. Calcification might be detected in the minority of cases. A lucent zone around SH, the "*air meniscus sign*", first described in 1978 [29], is a typical radiological feature representing trapped air around the lesion. Additionally, other reports of air spaces surrounding SH have been published [30]. However, other diagnoses must be considered, including carcinoids, hamartoma, hemangioma, malignant teratoma, arterio-venous malformations and inflammatory lesions. In the present case, chest radiography was normal, but thoracic computed tomography revealed a small but well-defined lesion of the left lower lobe with homogeneous contrast enhancement (Figure 1b). No typical lucent zone was found at the periphery of the lump, and no regional lymph node enlargement was present. Due to the history of metastatic CRC, however, a pulmonary spread of rectal cancer was the most probable diagnosis, so surgical resection of the lesion was performed.

During surgical intervention, we found early stage SH. Wedge resection in previous cases of early stage SH was associated with excellent long-term survival and therefore should be the treatment of choice if an exact pre- or intraoperative diagnosis is possible [3,31]. Otherwise, especially in cases of uncertain intraoperative frozen section examinations and given the uncertainty of growth, biological behavior, local recurrence and metastatic spread, the optimal therapeutic approach remains undefined. In these cases, atypical or anatomic resection with systematic lymphadenectomy is suggested [31]. Because of our patient's distinctive history, we oriented our therapy toward a strong suspicion of a pulmonary metastasis of CRC and elected to pursue a thorough surgical approach with atypical resection followed by regional lymphadenectomy [20].

Only a few cases of SH have been reported in young patients, among them a 10-year-old, an 18-year-old and a 19-year-old Asian female as well as a 22-year-old male, who presented with lymph node metastases implying a more malignant case of SH [12,32]. The latter might corroborate with the monoclonality of cells within SH, which has been described before and which suggests a neoplastic growth pattern of the lesion [33]. With respect to synchronous colorectal neoplasms, female patients suffering from FAP and simultaneous SH have been described [13,14]. In these cases, patients did not have any extracolonic manifestations of FAP and did not suffer from CRC until they presented with SH. To the best of our knowledge, this is the first report of SH associated with Lynch syndrome.

Autosomal-dominant Lynch syndrome (HNPCC) is a rare genetic disease (OMIM #609310) that usually shows right-sided predominance of CRC at a young age and is often caused by mutations of MMR-genes [34]. Although occurrence is less frequent than CRC there is a high prevalence of synchronous or metachronous extracolonic manifestations, especially endometrial cancer, which caused the death of our patient's mother. Other extracolonic manifestations include gastric, genitourinary, ovarian, small bowel, brain and sebaceous tumors [34,35]. Only one case of Muir-Torre syndrome, a variant of Lynch syndrome with additional skin lesions, was reported that was associated with non-small cell lung cancer [36]. However, there are no reports of benign lung tumors as extracolonic manifestation of Lynch syndrome.

In our patient, MSI testing of SH and immunohistochemistry for MLH1, MSH2, MSH6 and PMS2 did not reveal MSI or loss of MMR-expression in the pulmonary nodule. On the one hand we would have judged SH as an extracolonic manifestation of Lynch syndrome in this specific patient if SH would have featured MSI and loss of MMR-expression. On the other hand, one might anticipate that high-grade MSI and loss of MMR-expression by homocygosity of a mutated PMS2 should then have led to a more malignant growth pattern of SH. Pulmonary SH, as in the present case (*Ki*-67 index <5%), is a mostly benign and heterogeneous tumor composed of different cell types and exhibits various histological patterns [33]. Nevertheless, heterozygosity of PMS2 in the present case as exhibited by c-DNA-sequencing might still be causally associated with the development of this exceedingly rare tumor. Although a sporadic coincidence of SH and Lynch syndrome could not be ruled out in our patient, one might raise the suspicion of a common etiology being responsible for the exceptional concurrence of these two extremely infrequent events in a young male Caucasian.

## Conclusions

We present the first case of pulmonary SH in a young Caucasian male and in a patient suffering from Lynch syndrome. It might be speculated that SH did not just incidentally co-occur with the patient's CRC. From this unlikely concurrence we assume that the underlying Lynch syndrome might have abetted the arising of the patient's SH and hypothesize a common cause for these rare events. However, SH could not be termed as an extracolonic manifestation of Lynch syndrome since it obviously showed a benign behavior and did not exhibit MSI or loss of MMR-expression based upon heterozygosity of PMS2.

## Consent

Written informed consent was obtained from the patient for publication of this case report and any accompanying images. A copy of the written consent is available for review by the Editor-in-Chief of this journal.

## Competing interests

The authors declare that they have no competing interests.

## Authors' contributions

TSS, PNK and AK collected all patient's history data with substantial contribution of WET, MAK, RAH and KWJ. TSS, PNK and AK drafted the manuscript with committed and dedicated review and discussion of WET, MAK, RAH and KWJ. DM prepared the histopathological data and figures including their review and evaluation. All authors contributed substantially to the patient's care and therapy. All authors read and approved the final manuscript.
